# Osteoporosis, Osteoarthritis, and Subchondral Insufficiency Fracture: Recent Insights

**DOI:** 10.3390/biomedicines12040843

**Published:** 2024-04-11

**Authors:** Shunichi Yokota, Hotaka Ishizu, Takuji Miyazaki, Daisuke Takahashi, Norimasa Iwasaki, Tomohiro Shimizu

**Affiliations:** Department of Orthopedic Surgery, Faculty of Medicine, Graduate School of Medicine, Hokkaido University, Sapporo 060-8638, Japan; falcon8863@gmail.com (S.Y.); iszhtk24@gmail.com (H.I.); takuzimiyazaki@gmail.com (T.M.); rainbow-quest@pop02.odn.ne.jp (D.T.); niwasaki@med.hokudai.ac.jp (N.I.)

**Keywords:** osteoarthritis, osteoporosis, subchondral insufficiency fracture, bone mineral density, osteoporotic fracture

## Abstract

The increased incidence of osteoarthritis (OA), particularly knee and hip OA, and osteoporosis (OP), owing to population aging, have escalated the medical expense burden. Osteoarthritis is more prevalent in older women, and the involvement of subchondral bone fragility spotlights its association with OP. Notably, subchondral insufficiency fracture (SIF) may represent a more pronounced condition of OA pathophysiology. This review summarizes the relationship between OA and OP, incorporating recent insights into SIF. Progressive SIF leads to joint collapse and secondary OA and is associated with OP. Furthermore, the thinning and fragility of subchondral bone in early-stage OA suggest that SIF may be a subtype of OA (osteoporosis-related OA, OPOA) characterized by significant subchondral bone damage. The high bone mineral density observed in OA may be overestimated due to osteophytes and sclerosis and can potentially contribute to OPOA. The incidence of OPOA is expected to increase along with population aging. Therefore, prioritizing OP screening, early interventions for patients with early-stage OA, and fracture prevention measures such as rehabilitation, fracture liaison services, nutritional management, and medication guidance are essential.

## 1. Introduction

Osteoarthritis (OA), a chronic degenerative and debilitating condition affecting the entire synovial joint, is characterized by hyaline articular cartilage damage, subchondral bone degradation, tissue hypertrophy, synovial hypervascularity, and tendon and ligament instability [1]. Hip (HOA) and knee (KOA) OA cause progressive walking disability, resulting in decreased activities of daily living (ADL) and increased social burden. OA is common in older adults; it causes pain, affects physical functionality and other aspects such as mental health, sleep, and work engagement, and even increases mortality risk [2,3,4]. With the population aging, the prevalence and progression of OA is increasing annually [5]. Globally, the incidence of OA has increased by 58% from 1990 to 2017 [6].

Although OA has no definite curative treatment, in many countries, arthroplasty is commonly performed for severe OA [7,8,9,10]. While technological advances in arthroplasty have led to good long-term clinical outcomes, some cases result in revision arthroplasty owing to the consequential increase in healthy life expectancy [11,12]. Concurrently, osteotomies, to correct alignment and morphological abnormalities for preventing OA progression, and arthroscopy have advanced as well [13,14,15,16]. However, these treatments inevitably result in complications such as intra- and post-operative fractures, correction loss, and nonunion [17]. Therefore, early intervention to prevent OA onset and progression and to predict prognosis are of considerable importance in an aging society.

The relationship between OA and osteoporosis (OP) has long been a debatable topic. High bone mineral density (BMD) has been implicated in OA pathophysiology [18,19,20], and OA and OP were initially considered incompatible and contrary pathologies. Conversely, osteoporotic fractures in patients with lower-extremity OA (HOA and KOA) and the development of OA progression in patients with OP are being reported increasingly [21,22,23]. Subchondral insufficiency fracture (SIF), which is based on bone fragility and often causes irreversible deformity and significant ADL loss, has gradually been clarified. This review summarizes the latest findings on the relationship between lower-extremity OA and OP and SIF to provide insights into the complex relationship between OA and OP and their relationship with bone remodeling abnormalities as well.

## 2. Methods

PubMed (The National Library of Medicine, Bethesda, MD, USA) searches were conducted between 2001 and 2024 using the following combined search terms: “osteoarthritis”, “osteoporosis”, “bone mineral density”, “osteoporotic fracture”, “subchondral insufficiency fracture”, and “spontaneous osteonecrosis of the knee”. Two authors (S.Y. and H.I.) independently screened the titles and abstracts of all search results. After comparing the references, they reviewed the full text of potentially eligible studies. Following a consensus meeting to discuss each full-text article, the final studies to be included were selected. In the case of disagreements, a third author (T.S.) was consulted. If there were papers with similar conclusions, the more recent one was selected. In addition, we cited papers belong to the previous century that we considered minimally necessary for understanding the pathology.

## 3. Osteoarthritis

### 3.1. Epidemiology

According to the Global Burden of Disease (GBD) Study 2021, in 2020, approximately 595 million people, accounting for 7.6% of the global population, had OA, which is an increase of 132.2% in total cases since 1990; moreover, the worldwide age-standardized rate of years lived with disability (YLDs) for individuals with OA was 255.0 YLDs per 100,000 patients, an increase of 9.5% from 1990 [24]. Among adults aged ≥ 70 years, OA was the seventh leading cause of YLDs. The number of OA patients continues to increase due to population growth and aging, and is expected to continue to grow steadily. Between 2020 and 2050, a substantial rise from 48.6% to 95.1% in OA cases worldwide is estimated to occur. Notably, regions in sub-Saharan Africa are expected to experience an alarming increase of over 200% in cases by 2050. Although there is global diversity in the occurrence, onset, and YLDs attributed to OA, the burden is on the rise in the majority of countries [25].

The prevalence of KOA has been studied in many meta-analyses, and the trend is the same: it is the most common form of OA and increases year by year [26,27]. According to the GBD Study 2021, compared with those in 2020, cases of KOA are projected to increase to 74.9% by 2050 [24]. Analyzing information obtained from the Korea National Health and Nutrition Examination Survey spanning from 2010 to 2013, Hong et al. found that the prevalence of KOA stood at 35.1% in a representative group of Korean adults aged ≥ 50 years. The highest prevalence, reaching 78.7%, was identified in women aged ≥ 80 years. Compared to patients aged 50–59 years, adults aged ≥ 80 years faced a significantly higher risk of KOA, with an 8.87-fold increase. 

HOA is caused by biomechanical loading, and it showed similar longitudinal changes to those of KOA, making its management very essential in an aging society. According to the GBD Study 2021, compared with those in 2020, cases of HOA are projected to increase to 78.6% by 2050 [24]. In the Framingham Study Community cohort, the age-standardized prevalence of radiographic HOA was 19.6%, and that of symptomatic HOA was 4.2% in individuals aged ≥ 50 [28]. In a recent meta-analysis, there were regional differences in the prevalence of HOA; the lowest was in Africa at 1.20%, followed by Asia at 4.26% and North America at 7.95%, and the highest was in Europe at 12.59%, but these did not vary by sex [29]. In a large-scale population of a nationwide cohort study, Research on Osteoarthritis/osteoporosis Against Disability in Japan, the incidence rate of radiographic HOA was 5.6/1000 person years and 8.4/1000 person years, and the progression rate was 2.2/1000 person years and 6.0/1000 person years in men and women, respectively [30]. 

In addition to gender and aging, recent studies have revealed racial, ethnic, and economic differences in the prevalence and progression rates of OA [31]. Callahan et al. found that black people exhibited a higher prevalence and severity of lower-extremity OA than did white people. Additionally, lower socioeconomic status and rural areas have a high incidence of lower-extremity OA [32]. Thus, OA is affected by various factors other than biomechanical factors. However, OA onset and progression associated with biomechanical loading are extremely important and remain the subject of extensive research. A Mendelian randomization study indicated that body mass index exerts a major causal effect on OA risk at weight-bearing joints [18]. Engaging in physically demanding occupations, particularly those involving heavy physical workloads, increases the risk of developing lower-extremity OA [33]. Schram et al. reported that movements such as heavy lifting, squatting, knee bending, kneeling, and climbing are associated with an increased risk of both KOA and HOA [34]. Consequently, interventions aimed at minimizing exposure to such tasks, preventing joint injuries, and optimizing body weight can potentially decrease the onset and progression of lower-extremity OA. 

The association between lower-extremity OA and biomechanical alignment has also been reported previously. In patients with HOA, a comparison with healthy controls revealed a tendency toward a supinated foot posture [35]. Conversely, those with KOA are often found to exhibit a pronated foot posture and flat feet [36,37]. These findings indicate the presence of condition-specific foot postures in HOA and KOA, highlighting the potential impact of lower-extremity alignment on osteoarthritic conditions. Beyond the alignment of the lower extremities, recent evidence indicates a significant association between spinal and pelvic biomechanics with the prevalence and progression of HOA and KOA [38]. Specifically, an increased anterior spinal tilt and alterations in lumbar lordosis, along with variations in pelvic tilt and obliquity, have been identified as factors contributing to the pathomechanics of HOA and KOA [39,40,41].

### 3.2. Current Topics of Lower-Extremity Osteoarthritis Treatment

Other than surgical treatment such as arthroplasty or osteotomies, current OA management emphasizes prioritizing exercise and physical therapy, using assistive devices such as canes or splints, making home modifications, and actively engaging in self-management educational programs [42,43]. Overton et al. reviewed the congruence of the various guidelines for OA and found that all of them emphasized physical treatment and therapy [44]. Osteoarthritis Research Society International (OARSI) summarized that core treatments for KOA, HOA, and polyarticular OA included arthritis education and structured land-based exercise programs [45]. Simic et al. reported that clinical factors independently associated with radiographic progression were the use of NSAIDs and not meeting physical activity guidelines [46]. The COVID-19 pandemic has influenced the development and implementation of web-based physical activity programs, especially for lower-extremity OA [47,48,49]. Nelligan et al. reported that an online self-directed strengthening exercise regimen and physical activity guidance supported by automated behavior change text messages to encourage exercise adherence demonstrated enhanced knee pain relief and improved function at the 24-week follow-up [50]. The lifestyle program “Plants for Joints” demonstrated a reduction in stiffness, alleviation of pain, and enhancement of physical function in individuals with metabolic syndrome-associated lower-extremity OA when compared with those under standard care [51].

Recent research on KOA has explored its association with trace elements and vitamin D. Studies have found that increased macronutrient loss, particularly of calcium, phosphorus, and magnesium, in the femoral neck correlates with specific groups of OA patients, including females aged 51–60 years and individuals over 70 years [52]. Additionally, investigations have revealed that a higher dietary intake of magnesium and potassium is linked to fewer OA symptoms and better quality of life [53]. Vitamin D has also garnered significant attention, with studies indicating that maintaining sufficient serum levels of vitamin D in KOA patients can lead to less cartilage volume loss and improved physical function [54,55,56,57]. However, conflicting results exist regarding the effects of vitamin D supplementation on knee pain and cartilage volume in KOA patients [58,59]. Thus, although there are many studies on vitamin D and KOA, no certain view has been reached. Because vitamin D sufficiency significantly affects bone metabolism and is an appropriate indicator for OP treatment, drugs such as active vitamin D3 analogues are often used in OP treatment [60]. Future studies on the relationship between KOA and vitamin D sufficiency, taking OP into account, may provide new insights. 

### 3.3. Subchondral Microenvironment of Osteoarthritis

OA causes substantial changes in bone density and microstructure in the joint micro-environment [61]. In early-stage OA, the subchondral bone plate undergoes thinning and increased porosity concomitant with initial cartilage degeneration. Simultaneously, subchondral trabeculae deteriorate, characterized by increased trabecular separation and diminished trabecular thickness [62,63,64]. Conversely, in late-stage OA, the subchondral bone plate and trabeculae increase in thickness. Despite the increased bone volume and elevated local bone turnover, reduced bone mineralization and a reduced elastic modulus of the bone tissue occur [65]. In addition, the subchondral cancellous bone may maintain in an osteopenic state [62]. Consequently, mechanical integrity is compromised, making the bone more susceptible and fragile to mechanical loading-induced deformation. In early-stage OA, microcracks are frequently observed in the thinned subchondral bone. Microcracks have a protective function in subchondral bone homeostasis and prevent extensive bone damage [66,67]. Thus, OA may be regarded as “subchondral micro fracture” caused by abnormalities and microcrack accumulation. Moreover, OA progression can be considered a continuous and over-repair process against micro-damage [68], resulting in degenerative changes such as spur and bone sclerosis in late-stage OA. On the other hand, excessively fragile bone may develop “subchondral macro fracture” instead of “subchondral micro fracture”. Thus, bone fragility has recently been recognized as a very critical point in understanding the subchondral environment of OA.

In summary, OA is affected by various factors, highlighting the necessity for a comprehensive treatment approach in addition to surgical intervention. In particular, OA and OP are not opposites, but are likely to be intricately intertwined via bone metabolism.

## 4. Osteoporosis

### 4.1. Epidemiology

OP is a common skeletal disease characterized by diminished BMD [69]. Consequently, patients with OP are at an elevated risk of osteoporotic fractures [70,71], which frequently lead to decreased ADLs and burden healthcare systems [72,73]. Population aging has increased the incidence of OP and consequent osteoporotic fractures. A recent meta-analysis reported that the global prevalence of OP and osteopenia was 19.7% and 40.4%, respectively [74]. The prevalence was greater in developing countries, and increased with age, regardless of sex. These trends were similar with those of OA. 

OP has a wide range of causes, but can be broadly divided into primary and secondary OP [75]. Primary OP includes postmenopausal OP, male OP, and idiopathic OP (such as post-pregnancy OP). Secondary OP is caused by rheumatoid arthritis, diabetes mellitus, chronic kidney disease, liver disease, and alcoholism, as well as endocrine, nutritional, drug-related (such as glucocorticoid-induced OP, etc.), immobility, and congenital causes, and is still under active investigation [76,77,78,79,80,81,82]. 

Postmenopausal OP is a condition where bones gradually weaken due to low estrogen levels and aging [83]. It is characterized as having excessive or imbalanced bone remodeling, leading to a progressive loss of bone mass and structure. Research suggests that nearly one-third of women aged ≥50 may experience osteoporotic fractures [84]. Previous studies indicate that estrogen levels are linked to BMD and help protect against osteoporotic fractures [85]. 

Male OP has been gaining attention recently. In recent decades, there has been a more pronounced increase in the prevalence of OP and fractures among males compared with that among females. While it is more commonly associated with women, it affects a significant portion of the male population, with about 12% worldwide and over 20% in certain regions [86,87]. Moreover, males experience higher morbidity and mortality from fractures compared with females [88,89]. In a Danish national cohort study, substantially higher mortality among male hip fracture patients than female hip fracture patients was observed despite the men being 4 years younger at the time of fracture [90]. 

### 4.2. Diagnosis

The diagnosis of OP is determined via a medical interview, physical examination, imaging (radiographs, dual-energy X-ray absorptiometry (DXA), magnetic resonance imaging (MRI), etc.), blood and urine tests, and differentiation from other diseases. In particular, the limitations of BMD evaluation via DXA have probably received the most attention in recent years. DXA is the established standard for assessing BMD [91]. However, its diagnostic sensitivity can be compromised by internal artifacts [92,93]. DXA provides a two-dimensional anteroposterior projection of the lumbar spine; hence, areal BMD measurements may be affected by structural irregularities, such as osteophytes and compression fracture-associated vertebral deformities [94]. This may lead to an overestimation of BMD, and consequently, a significant underestimation of OP risks. 

Radiofrequency echographic multi-spectrometry (REMS) is an emerging innovative non-ionizing technology for BMD assessment. It is radiation-free, portable, and cost-effective. REMS analyzes raw radiofrequency ultrasound signals acquired during an echographic scan of the lumbar spine and proximal femur, capturing detailed tissue property data typically overlooked in conventional B-mode imaging [95]. European studies have confirmed the diagnostic precision and reliability of REMS versus DXA [96,97]. An Italian study conducted on a white female population demonstrated that REMS effectively addressed common DXA artifacts, such as OA and vertebral fractures [98]. Ishizu et al. found that proximal femur BMD measurements obtained via REMS were significantly lower than those obtained via DXA, while a significant correlation was observed between the HOA Kellgren–Lawrence grade and proximal femoral T-score discrepancies. These findings suggested that REMS might not be influenced by artifacts and could more precisely evaluate bone fragility [99]. In the future, modalities that are less affected by artifacts, such as REMS, should be used for effective diagnosis.

### 4.3. Treatment

The identification of crucial pathways regulating bone resorption and formation has ushered in various treatment approaches. The primary goal of OP treatment is to increase BMD and prevent osteoporotic fractures. Anti-resorptive drugs, including bisphosphonates (BPs), selective estrogen receptor modulators, and denosumab, have been acknowledged for their efficacy. Osteoanabolic drugs, such as teriparatide, romosozumab, abaloparatide, and strontium (Sr) ranelate, should be considered for patients with an extremely high or imminent fracture risk. However, because of the restricted treatment duration of 12–24 months with most osteoanabolic drugs, long-term anti-resorptive drugs are recommended [100,101]. Adequate treatment strategies for individuals with high fracture risk remain a challenge, highlighting the importance of the widespread implementation of fracture liaison services (FLS) and improved adherence to therapy in the future [102].

### 4.4. Pathogenesis

In the normal bone environment, there exists an equilibrium between bone formation by osteoblasts and bone resorption by osteoclasts, maintaining bone mass and BMD. This equilibrium is disrupted in OP, resulting in bone resorption surpassing bone formation due to either the inhibition of osteoblasts or the heightened activity of osteoclasts [103]. OP causes a decrease in bone strength due to a combination of factors, not only including decreased BMD resulting from abnormal bone remodeling, and but also decreased bone quality, caused by structural deterioration, decreased secondary calcification, increased oxidative stress and glycation, changes in bone matrix proteins due to vitamin D and vitamin K deficiency, etc. [104]. Moreover, various cytokine networks and signaling pathways are known to be involved in the pathogenesis of OP [105], and the pathogenesis of OP varies in detail from individual to individual.

In summary, although the pathogenesis of OP has been extensively researched in a wide variety of conditions and various therapeutic agents have been developed, it is a disease that should continue to be studied in the future. It is essential to assess BMD more accurately and to understand the pathophysiology in each case in order to determine a treatment plan.

## 5. Relationship between Osteoarthritis and Osteoporosis

### 5.1. High Bone Mineral Density and Osteoarthritis

Several studies have reported on the association between high BMD and lower-extremity OA [20,106,107]. In the UK Biobank study, evidence of the causality of all OA, KOA, and HOA was observed for high femoral neck BMD [18]. Zamzam et al. found that BMD, right femoral neck T-score, and BMI can be used as predictors for KOA development and progression [108]. Cai et al. reported that medial subchondral BMD and systemic BMD were positively associated with an increased risk of total knee arthroplasty (TKA) and total hip arthroplasty (THA), respectively, suggesting the role of BMD in OA progression [109]. Conversely, some reports revealed that a high BMD was not clearly associated with KOA progression, although it was positively associated with osteophytes [110]. Notably, the aforementioned reports were mostly based on DXA, which may be an inaccurate assessment of BMD. Evidence surrounding association between high BMD and higher-extremity OA is summarized in Table 1.

### 5.2. Low Bone Mineral Density and Osteoarthritis

The association between OP, low BMD, and OA has increasingly been reported [19,74,111]. Heiss et al. used quantitative CT to assess BMD at the femur and tibia, cortical bone plate, and epiphysis in three locations (subchondral, mid-epiphyseal, and juxtaphyseal) in 275 patients with KOA [112]. Patients with Kellgren–Lawrence grade 2–4 KOA had low BMD in the medial femoral condyle at all epiphyseal locations, and BMD decreased with increasing distance from the joint. Yoo et al. observed that patients with OP and osteopenia had a tendency of accelerated OA progression and an increased likelihood of requiring surgical intervention. They posited that low BMD may contribute to subchondral microfractures [113]. Lower-extremity OA, especially in postmenopausal women, is complicated by OP. Stamenkovic et al. demonstrated that in postmenopausal women, the BMD grade and T-score of the spine and femoral neck are low in more severe forms of OA [23]. Postural changes identified in postmenopausal women have been associated with bone loss and joint degeneration [114]. Thus, low BMD might influence OA onset and progression.

Many studies have reported on OP screening and treatment with arthroplasty. In one study, among 109 patients who underwent TKA, 19 patients (17.4%) and 50 (45.9%) patients had OP and osteopenia, respectively [115]. Drees et al. reported an extremely high occurrence of low BMD among patients who underwent TKA or THA, especially males; 20% and 38.8% had OP and osteopenia, respectively, compared with the general population [116]. Evidence surrounding the association between low BMD and lower-extremity OA is summarized in Table 2.

### 5.3. Osteoporotic Fracture and Osteoarthritis

Many studies have reported on the increased susceptibility to osteoporotic fracture in patients with lower-extremity OA, which is considered dangerous. Studies have revealed decreased BMD in the affected femoral neck in patients with KOA [21,117]. A retrospective study including 250,000 patients reported that after a 10-year follow-up, 12.1% of patients with OA had at least one fracture, a significantly higher percentage than that in those without OA (7.7%) [22]. However, osteoporotic fracture in patients with lower-extremity OA might be complicated by various co-factors, including medications (proton pomp inhibitors, anti-depressants, or sleeping pills) [118,119,120,121], worsening joint range of motion and walking disability. 

### 5.4. Osteoporosis Treatments and Osteoarthritis

The efficacy of OP drugs for OA has been well reported. In a mouse model of KOA, parathyroid hormone (PTH, 1–34) demonstrated cartilage-protective effects by reducing wear, preserving thickness, and maintaining glycosaminoglycan levels [122]. In an ovariectomized mice model of OA, 8 weeks of 17β-estradiol (E2) treatment prevented cartilage damage and synovial thickening, and motor activity was improved after 2 weeks, which was associated with lower pain sensitivity in the OA paw. Furthermore, E2 treatment protected against ovariectomy-induced loss of subchondral trabecular bone [123]. While zoledronate acid improved clinical symptoms and delayed THA in patients with HOA with increased bone turnover [124], some studies have reported its non-protective effect against KOA [125]; hence, its protective effects remain inconclusive. Sr ranelate has been widely reported for its efficacy against OA [126,127]. Reginster et al. showed that Sr ranelate was associated with smaller reductions in joint space width, fewer radiological progressors, and greater reductions in the WOMAC score [128]. In monoiodoacetate-induced OA rats, the prophylactic and therapeutic administration of Sr ranelate was associated with improved pain and joint discomfort [129]. Han et al. indicated that the impact of Sr ranelate on subchondral bone suggests its potential as a disease-modifying OA drug (DMOAD) for managing OA [130].

In summary, although both high and low BMD are involved in OA, considering the association with osteoporotic fractures and the effect of OP drugs, it is inferred that bone fragility has no small effect on OA.

## 6. Subchondral Insufficiency Fracture of the Knee

### 6.1. Epidemiology

Spontaneous insufficiency fracture of the knee (SIFK), first described by Ahlback in 1968 as spontaneous osteonecrosis of the knee (SONK), is characterized by a sudden onset of knee pain [131]. Yamamoto et al.’s histological study clarified that SONK was a subchondral fracture and that osteonecrosis occurred secondarily post-fracture [132]. This has been confirmed via MRI imaging, and it is currently accepted that SONK represents the end stage of SIFK [133]. SIFK is an uncommon condition, with a reported prevalence of 2.94% among 340 cases of symptomatic knee conditions diagnosed with MRI [134]. Numerous studies show that SIFK is more common in people over 50–60 years, and that it is observed more often in women than in men [135,136,137,138,139,140]. Pelet reported that out of 73 SIFK patients, 47 (64.4%) were women, with an average age of 62.8 years for all patients [141]. The medial femoral condyle (64.9%) is the most common site in SIFK, followed by the medial tibial plateau and lateral femoral condyle [137]. Patients usually describe a sudden onset of mostly unilateral pain occurring non-traumatically, which is disproportionate to changes observed on radiography [142,143].

### 6.2. Diagnosis and Progression

In the initial stages of SIFK, plain radiography findings are often normal, limiting the diagnostic value of radiographic imaging in early-stage SIFK detection [139]. However, radiographic abnormalities such as femoral condylar flattening, osteochondral defects, or epiphyseal deformities may be identified in later stages [144]. MRI, with its high-quality imaging of osteochondral lesions, is the most optimal modality for the early diagnosis of SIFK [133]. Characteristic MRI findings include a subchondral hypointense line and bone marrow edema-like hyperintense lesion on T1- and T2-weighted images, respectively, of the affected condyle [133,145]. Furthermore, initial radiographic findings demonstrate mild radiolucency [139]. Progressive SIFK can result in subchondral collapse, culminating in end-stage OA and necessitating surgical intervention [137,146].

### 6.3. Treatment

Conservative management is the primary treatment approach for SIFK, particularly in the early stages and in the absence of accompanying meniscal injuries [147]. Current non-operative management strategies for SIFK include observation, nonsteroidal anti-inflammatory drugs, non-weight-bearing practices, and the use of wedge insoles [138,148]. However, no randomized trials have addressed weight-bearing status and rehabilitation protocols in SIFK. Other medications reportedly have limited efficacy. Joint replacement surgery remains the standard surgical treatment for SIFK, especially in cases of advanced or end-stage arthritis, or when joint-preserving treatments have failed [149]. Unicompartmental knee arthroplasty (UKA) may be suitable for patients with degenerative changes limited to a single compartment, as it preserves cartilage in the other compartments [150,151]. In cases where the pathological process extends to the other compartments, or in severe SIFK-induced OA, TKA is the predominant and reliable treatment choice [152,153]. At stages where joint degeneration is assessed as mild to moderate, a selection of joint-preserving surgical options is considered viable. High tibial osteotomy (HTO), which shifts the pressure from the degenerated part of the knee joint to the healthy part, is a useful treatment option compared with UKA and TKA, especially in younger patients, for joint preservation and enhanced subchondral bone healing [154,155]. Other joint-preserving treatments for pre-collapse SIFK include core decompression to alleviate pressure at the damaged site [156], arthroscopic microfracture to promote the repair of the injury site [157], and osteochondral autografting [158] and subchondroplasty [159,160] to fill the damaged area a osteochondral graft and engineered calcium phosphate compound, respectively.

These joint-preserving procedures have been shown to decelerate the progression of degenerative changes in joints, thereby producing favorable outcomes in the short term. However, it remains a significant challenge to fundamentally prevent joint destruction and consequently avoid the necessity for joint arthroplasty. Consequently, for the fundamental suppression of joint destruction, it is crucial to develop therapeutic approaches to inhibit onset of SIFK by targeting the initial stage of pathophysiological changes.

### 6.4. Risk Factors and Pathogenesis

An age > 60 years, female sex [137,145,161], and the presence of conditions such as osteopenia or OP [132,142,162] have been extensively reported as risk factors for SIFK. Degenerative meniscal injuries [163,164] and cartilage damage resulting from pre-existing OA [145] can lead to an increased load in the medial compartment, potentially inducing SIFK. Joint collapse and progressive degenerative changes in SIFK often lead to severe OA, frequently necessitating TKA. In addition to older age and female sex [165], other risk factors for SIFK progression include lesion size [166,167], meniscal damage and OP, and knee varus alignment [145,168]. Meniscal injury and knee varus alignment can lead to increased contact pressure at the fracture site, ultimately resulting in joint collapse [165]. 

The pathophysiology of SIFK is not fully understood due to the rareness and the challenges of diagnosis at an early stage. However, considering these characteristics, subchondral bone fragility, lesion severity, and mechanical overload may be associated with the pathogenesis of SIFK. Micro-fracture is seen in subchondral bone fragility in the early phases of OA [66,67]. This finding implies that SIFK may represent a more severe manifestation of these microfractures, under conditions of more vulnerability and mechanical stress in the subchondral bone.

In summary, SIFK is likely a severe form of OA that is based on the fragility of subchondral bone. For its fundamental treatment, the control of bone fragility may be a new potential therapeutic target for the onset and progression of SIFK.

## 7. Subchondral Insufficiency Fracture of Femoral Head

### 7.1. Epidemiology

Subchondral fracture of the femoral head (SIFFH), first described by Bangil et al. in 1996, has been defined as a subchondral fracture of the femoral head associated with OP without any evidence of osteonecrosis of the femoral head (ONFH) [169]. Although SIFFH has been reported to occur as a fatigue fracture in healthy younger adults [170] and renal transplant recipients [171], SIFFH is commonly observed in older women with OP [172]. SIFFH was reported to be observed in 3.6% (460 out of 7349) of patients with a preoperative diagnosis of HOA and in 11.1% (41 out of 369) of patients with a diagnosis of ONFH [173]. Yamamoto et al. showed that 64.1% (25 out of 39) of the patients diagnosed with SIFFH from 2001 to 2011 were women, and the mean age was 66.3 years [174]. Shimizu et al. observed that 80.4% (33 out of 41) of the patients diagnosed with SIFFH from 2010 to 2019 were women, and the mean age was 61.6 years [175]. The most common symptom of SIFFH is an acute onset of severe hip pain, typically occurring non-traumatically [172]. While in some cases the hip pain resolves within a few months after onset, in other cases, it pain gradually worsens [176].

### 7.2. Diagnosis and Progression

Radiographs are usually unremarkable in patients with early-stage SIFFH [176]. The most distinctive MRI finding in SIFFH is a band-like hypointense lesion on T1-weighted imaging [173]. This band-like lesion, which histopathologically corresponds to a fracture line, is essential for SIFFH diagnosis [177]. SIFFH can be differentiated from ONFH by the shape of the band on T1-weighted images; in SIFFH, the band is convex toward the articular surface and parallel to the subchondral bone plate [178]. Additionally, on gadolinium-enhanced images, the region between the band and the articular surface typically exhibits a hyperintense signal [179].

In some cases, SIFFH may heal without further fracture progression, exhibiting sclerotic changes due to callus formation within several months [176]. However, in other cases, it may progress to collapse, accompanied by severe pain, requiring surgical intervention [173]. Furthermore, SIFFH may trigger the onset of rapidly destructive coxopathy (RDC), which is characterized by the rapid destruction of the hip joint within 6–12 months, accompanied by an increased inflammatory response [180,181].

### 7.3. Treatment

Conservative management, including rest, non-weight-bearing, the use of crutches, and traction, may be useful in preventing SIFH progression, and these are often initially recommended for patients without evidence of femoral head collapse [169]. However, reports that provide specific guidance on non-operative management such as strict non-weight-bearing or regimen duration are lacking. In cases of femoral head collapse, particularly in older patients, THA is strongly considered, with excellent outcomes anticipated [172,182,183]. The effectiveness of anterior rotational osteotomy, a joint-preserving surgery designed to shift the lesion away from the load-bearing area, has been reported in younger patients, especially those in the pre-collapse stage of the joint [184,185]. Other joint preservation techniques, such as core decompression with bone void filler rafting to support the subchondral-affected region [186] and bioabsorbable stabilization, which is internal fixation using hydroxyapatite poly-lactate acid-threaded pins [187], have been reported in pre-collapse SIFFH. Like its application in knee joints, subchondroplasty, which involves filling the damaged area with calcium phosphate, may also be applicable to SIFFH [188]. However, the limitations of these studies, which include unclear measurement sites of the lesion and unclear timing of assessments during SIFFH progression, small sample sizes, and short follow-up periods need to be noted.

In the early stages prior to collapse, conservative treatment, mainly non-weight-bearing, and joint-preserving surgeries may be considered as first options. However, in the hip joint, which bears the greatest load within joints of the human body, the effectiveness of these approaches is inevitably limited. Consequently, THA has become the fundamental treatment option in practice. Therefore, the development of a fundamental treatment is necessary to prevent onset and progression of SIFFH for the preservation of the healthy joint.

### 7.4. Risk Factor and Pathogenesis

SIFFH is common in older women with OP, suggesting that OP-induced bone fragility could be considered a primary cause of SIFFH [172,176]. SIFFH has been observed in patients with alkaptonuria [189], renal [171] or liver [190] transplants, and systemic lupus erythematosus [191,192], suggesting that bone fragility associated with these conditions may influence SIFFH onset. Conversely, a study reported no significant differences in BMD between patients with SIFFH and controls [193]. However, BMD measurements may be affected depending on the anatomical location and disease stage [94]. The absence of changes in BMD does not negate the association between SIFFH and bone fragility.

Morphological abnormalities of the hip joint such as posterior pelvic tilt [194], hip dysplasia [193], and an inverted acetabular labrum [195,196] are observed in patients with SIFFH. These factors may contribute to the development of SIFFH by increasing the load on the subchondral bone of the femoral head. Notably, SIFFH can progress to advanced OA or RDC due to femoral head collapse, ultimately necessitating THA [181,182,197]. Older age [178], female sex [174], the location [198] and length [199] of the fracture line as observed on MRI, biomarkers of joint space narrowing [175], pelvic tilt [200], and pelvic range of motion [201] have been identified as risk factors for SIFFH progression. Thus, subchondral bone fragility, fracture severity, and mechanical overload may be involved in SIFFH pathogenesis.

The pathophysiology of SIFFH remains unclear partly due to the difficulty of diagnosis at an early stage prior to collapse. However, these findings suggest that mechanical excessive stress against the vulnerable subchondral bone of the largest joints may play a crucial role in the pathophysiology of SIFFH. Given the involvement of subchondral microfractures due to fragility in the onset of OA, SIFFH can be considered a more severe manifestation of OA, similar to SIFK.

In summary, the onset and collapse of SIFFH is likely associated with subchondral bone fragility. The effectiveness of treatments, other than THA, is limited due to the high risk of collapse. Therefore, exploring new approaches aimed at improving the fragility of the subchondral bone could potentially offer a strategy to prevent the occurrence of SIFFH.

## 8. Discussion

This review summarizes the relationship between lower-extremity OA and OP, including recent studies on SIF. Micro-damage and bone metabolic change in subchondral bone influence OA development [68,202,203]. SIF can provide a more noteworthy background to understand OA pathogenesis. Alterations in whole-body alignment and range of motion, including changes in foot posture, as well as spinal and pelvic posture, have impacts on lower-extremity OA [37,38]. Improper pressure induced by these pathological alignments may facilitate the localized accumulation of microdamage, potentially leading to SIFFH and SIFK. The etiology of both SIFK and SIFFH involves subchondral bone fragility [162,172] and distinctive morphological characteristics such as meniscal tears [167], an inverted acetabular labrum [195,196], knee varus alignment [168], hip dysplasia [193], and pelvic tilt [200]. Fragility, morphology, and fracture size accelerate collapse, leading to articular surface deformities. In such cases, SIF progresses to secondary OA accompanied by joint space narrowing and incongruity, resulting in irreversible degeneration [175]. Additionally, SIFFH can induce RDC via an increased inflammatory response against the fracture fragments rather than collapse, resulting in extensive femoral osteolysis [180,181]. In the case of severe joint degeneration or destruction, arthroplasty remains the only therapeutic option [152,183]. Owing to the limitations of the efficacy of joint-preserving procedures [172,204] and the revision surgeries required in cases of arthroplasty complications [11,12], the treatment strategy for SIF and OA should be carefully considered at an earlier stage via early diagnosis, based on an appropriate assessment of the risk of joint collapse. Subchondroplasty has been reported to show promising short-term outcomes as a treatment for subchondral pathology [159,160,188], suggesting it could be considered a therapy to reinforce the strength of the subchondral bone in the short term and locally. However, it is anticipated that it might not fundamentally prevent the decline in bone strength over time, due to untreated enhanced bone resorption at the subchondral bone resulting in fragility.

**Figure 1:**
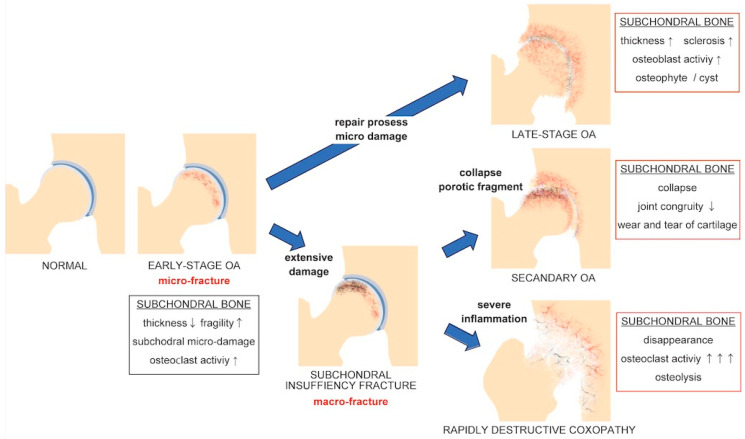
Mechanism of joint deterioration. All joint deterioration cases begin with micro-damage to the subchondral bone. Late-stage osteoarthritis (OA) develops as a reparative response to micro-damage. Subchondral insufficiency fractures (SIFs) are characterized by extensive subchondral damage. SIF can progress to secondary OA due to collapse. Severe inflammatory response to fracture fragments leads to rapidly destructive coxopathy. ↑, increase; ↑↑↑, dramatic increase; ↓, decrease.

A bone remodeling-mediated subchondral micro-environment is crucial to elucidating SIF And OA pathophysiology. Distinct microstructural changes in the subchondral bone are evident at different OA stages [68,202,205]. In early-stage OA, the subchondral bone plate thins and becomes more porous and vulnerable, characterized by dominant osteoclast-mediated bone resorption [206,207,208]. Conversely, in late-stage OA, the subchondral bone plate thickens and scleroses, characterized by dominant osteoblast-mediated bone formation [209,210,211]. In early-stage OA, microcracks are frequently observed in the thinned subchondral bone. Microcracks have a protective function in subchondral bone homeostasis and prevent extensive bone damage [66,67]. Thus, OA may be regarded as “subchondral micro fracture” caused by abnormalities and microcrack accumulation. This concept is consistent with the high prevalence of OA in postmenopausal older women [212,213] and the effectiveness of OP treatment against OA progression in mouse models [122,123]. Moreover, because OA progression can be considered a repair process against micro-damage [68], SIF can be considered “subchondral macro fracture” that exceeds the repair capacity of the osteoblasts. The subchondral microenvironment plays an important role in the pathophysiology of each joint disease, as illustrated in Figure 1. As mentioned, an incorrect initial response to SIF can lead to rapid irreversible degenerative changes. Thus, OA and OP are closely related, and the concept of OP-related OA (OPOA) requires widespread dissemination. The epidemiological trends of OP and an aging society may lead to increased OPOA incidence in the future.

Despite numerous reports linking OP to OA and SIF, a consensus remains elusive. Furthermore, several reports have associated high BMD with OA [20,106,107,108,109]. However, these findings may be indicative of subchondral sclerosis in late-stage OA, with decreased rigidity reportedly caused by insufficient mineralization in the sclerotic subchondral bone [214]. In addition, the subchondral trabecular bone may be osteopenic. A high BMD, sometimes observed in OA, could result due to abnormal bone remodeling in response to subchondral lesions, and may not constitute evidence for the relationship between OA and OP. Moreover, BMD measurements via DXA, which are susceptible to OA-related artifacts, often exhibit high values, while possibly masking bone fragility. While KOA has been reported to be associated with low femoral neck BMD in recent years, the few reports suggesting an association with HOA resulted the estimation that artifacts such as degenerative changes in HOA could affect high femoral neck BMD in DXA. Therefore, some patients with OA and a high BMD may have occult OPOA. 

The effects of various OP treatments in patients with OA have been well researched. Research on estrogen preparations aiming to prevent OA progression and improve its symptoms is ongoing. Other studies have investigated PTH formulations and BPs, which consistently exert a protective effect on cartilage and subchondral bone and maintain subchondral trabecular bone volume. Moreover, OP medications may be beneficial in preventing OA and SIF progression [215,216,217]. The aforementioned positive effects of OP treatments on lower-extremity OA are consistent with the understanding that OP and SIF affect OA. OP treatment might positively influence the subchondral micro-environment involved in OA by improving abnormal bone remodeling. In the future, OP screening and treatment for both patients with early-stage OA and those who are asymptomatic may help reduce OA incidence. Furthermore, the susceptibility of patients with OA to osteoporotic fractures remains a major issue. In this regard, existing OP-targeted rehabilitation and training programs and new measures such as FLS, nutritional management, and medication guidance may have positive effects.

Despite numerous reports linking OP to OA and SIF, a consensus remains elusive. Furthermore, several reports have associated high BMD with OA [20,106,107,108,109]. However, these findings may be indicative of subchondral sclerosis in late-stage OA, with decreased rigidity reportedly caused by insufficient mineralization in the sclerotic subchondral bone [214]. In addition, the subchondral trabecular bone may be osteopenic. The high BMD sometimes observed in OA could have resulted due to abnormal bone remodeling in response to subchondral lesions, and may not constitute evidence for the relationship between OA and OP. Moreover, BMD measurements via DXA, which are susceptible to OA-related artifacts, often exhibit high values, while possibly masking bone fragility. Therefore, some patients with OA and a high BMD may have occult OPOA. 

The effects of various OP treatments in patients with OA have been well researched. Research on estrogen preparations aiming to prevent OA progression and improve its symptoms are ongoing. Other studies have investigated PTH formulations and BPs, which consistently exert a protective effect on cartilage and subchondral bone and maintain subchondral trabecular bone volume. Notably, Sr ranelate significantly reduces subchondral bone remodeling and inhibits articular cartilage degeneration [218]. Sr ranelate enhances bone formation without triggering increased bone resorption, serving as an “uncoupling agent” that positively influences overall bone metabolism and potentially benefits OA pathophysiology [219]. Moreover, OP medications may be beneficial in preventing OA and SIF progression [215,216,217]. The aforementioned positive effects of OP treatments on lower-extremity OA are consistent with the understanding that OP and SIF affect OA. OP treatment might positively influence the subchondral micro-environment involved in OA by improving abnormal bone remodeling. In the future, OP screening and treatment for both patients with early-stage OA and those who are asymptomatic may help reduce OA incidence. Furthermore, the susceptibility of patients with OA to osteoporotic fractures remains a major issue. In this regard, existing OP-targeted rehabilitation and training programs and new measures such as FLS, nutritional management, and medication guidance may have positive effects.

## 9. Conclusions

OA causes abnormalities in subchondral bone and bone remodeling, closely related to OP. The novel concept that OA is affected by subchondral vulnerability and SIF, resulting in joint collapse and incongruity, leading to OPOA, merits attention. Because the prevalence of OPOA may increase in an aging society, OP screening and treatment for patients with early-stage OA or healthy patients are urgently needed.

## 10. Future Directions

Elucidating the mechanisms of osteoporotic subchondral fragility as the etiology of OA and the establishment of new diagnostic tools that can more accurately assess bone vulnerability can be expected to enable stage-specific fundamental treatments for OA, SIF, and OP.

## Figures and Tables

**Table 1:** Studies evaluating the onset or progressive factors of osteoarthritis focused on high bone mineral density.

Author, Year	n	Sex	Instruments for BMD Measurement	KOA or HOA	Onset Factors	Progressive Factors
Bergink, 2019 [106]	4154	Both	NR	Both	High FN BMD	NR
Funck-Brentano, 2022 [18]	384,838	Both	QUS (calcaneus) MR analyses	Both	High FN BMD (According to MR Analyses) BMI, Low Systolic BP	NR
Zamzam, 2023 [108]	487	Female	DXA	KOA	High FN BMD Age, Weight	High FN BMD
Cai, 2020 [109]	1095	Both	DXA	KOA (TKA)	High Medial Tibial Subchondral BMD	NR
				HOA (THA)	High Systemic BMD, High LS BMD	NR
Hartley, 2020 [110]	169	Both	DXA	KOA	High Bone Mass (TH, LS, or Systemic), Age	NR

Abbreviations: BMD, bone mineral density; NR, not reported; QUS, quantitative ultrasound; MR, Mendelian randomization; DXA, dual-energy X-ray absorptiometry; KOA, knee osteoarthritis; HOA, hip osteoarthritis; FN, femoral neck; BMI, body mass index; BP, blood pressure.

**Table 2:** Studies evaluating the onset or progressive factors of osteoarthritis focused on low bone mineral density.

Author, Year	n	Sex	Instruments for BMD Measurement	KOA or HOA	Onset Factors	Progressive Factors
Choi, 2023 [111]	149	Both	DXA	KOA	Low FN BMD	NR
Heiss, 2023 [112]	275	Both	Quantitative CT	KOA	Low Medial Femoral Condyle BMD	NR
Yoo, 2023 [113]	2492	Both	NR	KOA	Age, Sex, BMI, Occupation	Low BMD Age, Sex, BMI
Stamenkovic, 2022 [23]	96	Female	DXA	KOA	Low LS BMD, Low TH BMD	NR
Delsmann, 2022 [115]	109	Both	DXA	KOA	Low BMD	NR

Abbreviations: BMD, bone mineral density; DXA, dual-energy X-ray absorptiometry; CT, computed tomography; NR, not reported; KOA, knee osteoarthritis; HOA, hip osteoarthritis; FN, femoral neck; BMI, body mass index; LS, lumbar spine; TH, total hip.
